# Multidrug-Resistant pESI-Harboring *Salmonella enterica* Serovar Muenchen Sequence Type 82 in Poultry and Humans, Israel, 2020–2023

**DOI:** 10.3201/eid3110.250191

**Published:** 2025-10

**Authors:** Janet Perry, Tal Rakler, Katya Arnold, Anat Wiseman, Cinthia Satuchne, Yaniv Pima, Galina Moiseeva, Ilana Maler, Eugenia Yakunin, Assaf Rokney, Ehud Elnekave

**Affiliations:** Hebrew University of Jerusalem Robert H Smith Faculty of Agriculture Food and Environment, Rehovot, Israel (J. Perry, T. Rakler, K. Arnold, E. Elnekave); Egg and Poultry Board, Re'em Masmiya, Israel (A. Wiseman, C. Satuchne, Y. Pima); Kimron Veterinary Institute, Beit Dagan, Israel (G. Moiseeva, I. Maler); State of Israel Ministry of Health, Jerusalem, Israel (E. Yakunin, A. Rokney)

**Keywords:** Salmonella Muenchen, salmonellosis, poultry, multidrug resistance, MDR, pESI plasmid, antimicrobial resistance, horizontal transmission, foodborne infections, bacteria, food safety, zoonoses, Israel

## Abstract

*Salmonella enterica* serovar Muenchen emerged in Israel in 2018 and became a major public health threat. We aimed to determine the role of poultry in rising human cases, transmission routes within the broiler industry, and genetic similarity to *Salmonella* Muenchen found globally. We used whole-genome sequencing to compare *Salmonella* Muenchen isolates from poultry, food, and humans collected in Israel (2020–2023; n = 109) and globally (n = 125). *Salmonella* Muenchen sequence type 82 isolates from Israel harbored pESI plasmid, exhibited high genetic similarity between human and poultry sources, and closely resembled international pESI-positive strains; we found quinolone-resistance determinants in 58.6% of isolates. *Salmonella* Muenchen prevalence in commercial broiler flocks was 61.5% (95% CI 51.5%–71.5%); strains could not be traced to breeder flocks, but on-farm persistence existed. The clonal spread of *Salmonella* Muenchen in poultry contributes to increased incidence in humans. Horizontal transmission in broilers requires control measures to protect public health.

Nontyphoidal *Salmonella* (NTS) is a major cause of foodborne illness worldwide (*1*); poultry serve as a reservoir and a source of infection to humans for multiple serotypes (*2*). Although most serotypes cause little harm to the infected bird (*3*), controlling NTS infection in poultry is essential for the protection of public health (*2*). Given the pyramidal structure of the poultry industry (a large number of production flocks [broilers and layers] are descendants of a small number of parent and grandparent breeding flocks), control measures for NTS spread, such as vaccination against specific serotypes (*2*), are often focused on the parent and grandparent flocks and not on the high number of short-lived production birds.

In Israel, NTS surveillance in humans relies on passive reporting of clinical cases to the national reference laboratory of the Ministry of Health (*4*), similar to the approach used in Europe (*5*). In the poultry sector, the Egg and Poultry Board and the Israeli Veterinary Services conduct an active surveillance program for *Salmonella* only in breeding flocks and layer hen farms, in accordance with international regulations (European Regulation no. 2160/2003).

Since 2018, *Salmonella* Muenchen and *Salmonella* Virginia, formerly misclassified as separate serotypes (*6*) (both referred to as *Salmonella* Muenchen hereafter), emerged as the dominant serotypes in clinical cases in humans in Israel. These serotypes accounted for 2.0% (101/5,072) of all *Salmonella* cases in 2017 and 35.4% (1,124/3,171) in 2022 (*7*). A similar trend was found in *Salmonella* isolates from poultry: from 3.8% (63/1,672) of the animal and poultry sources in 2017 to 23.6% (202/857) in 2021, and from 4.9% (12/245) of the food industry and ready-to-eat products (most of which are from poultry slaughterhouses) in 2017 to 20.0% (43/215) in 2021 (*7*).

Another study in Israel (*8*) reported that *Salmonella* Muenchen had acquired pESI, a plasmid first identified in *Salmonella* Infantis (*9*). pESI is described as a mega-plasmid that contributed to stress tolerance and increased pathogenicity that enabled the rapid spread of *Salmonella* Infantis in humans and poultry in Israel since 2007 and later in Italy (*10*) and other locations worldwide (*11*). The spread in Israel was ultimately controlled by the introduction of mandatory vaccination against *Salmonella* Infantis in breeder flocks in 2011 (*4*). However, despite a rapid reduction in the prevalence of *Salmonella* Infantis in breeder flocks, the success of the vaccination program to reduce the prevalence in broiler flocks was only evident several years after its initiation (A. Wiseman, pers. comm., telephone, 2020 Jul 1). Therefore, we suspected that the persistence of *Salmonella* Infantis in the farm environment and horizontal transmission between flocks, rather than vertical transmission from parent flocks, played a substantial role in its spread.

We used a large collection of *Salmonella* Muenchen isolates recovered from broiler flocks in Israel to determine the genetic relatedness among isolates from broilers, humans, and other poultry sources and the presence of relevant genetic determinants such as pESI and antimicrobial resistance (AMR) genes. We also determined the prevalence of *Salmonella* Muenchen in commercial broiler flocks and the potential transmission routes between different growth stages to observe whether vertical or horizontal transmission play a major role in bacterial spread. Finally, we studied genetic similarities between the emerging *Salmonella* Muenchen in Israel and global strains to reflect on the potential for its global spread and the risk posed to public health globally.

## Materials and Methods

### Study Population and Sample Collection

**Figure 1:**
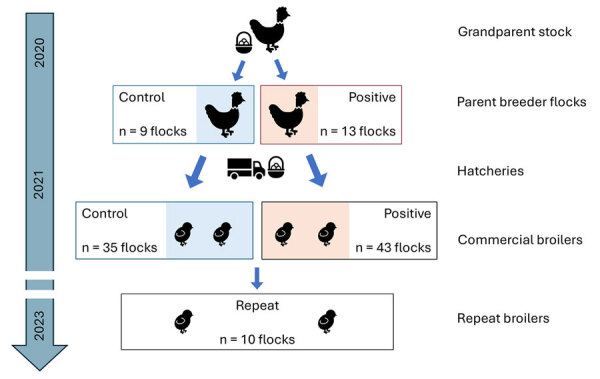
Timeline showing the selection of the positive and control breeder flocks for study of *Salmonella enterica* serovar Muenchen in poultry and humans, Israel, 2020–2023. We sampled commercial broiler flocks that were direct descendants of the positive and control breeder flocks during 2020–2021 and detected *Salmonella* Muenchen infection. We resampled a subset of the barns housing new broiler flocks during 2023 (repeat broilers).

We conducted 3 analyses of *Salmonella* Muenchen isolates (Appendix 1). We chose study populations and collected samples in accordance with the intention of each analysis. For analysis A, we identified *Salmonella* Muenchen isolates collected in Israel during June 2020–January 2022 from a broad geographic distribution from broilers (n = 60; see analysis B) and layers (n = 9), poultry slaughterhouses (n = 9) (Appendix 1 Figure 1), and human clinical cases (n = 21). For analysis B, we used isolates from heavy breeders, grandparent flocks and hatcheries that were collected as part of the national *Salmonella* active surveillance program and were tested for *Salmonella* in accordance with International Standards Organization protocol ISO-6579–1 (*12*). Subsequently, we screened all serogroup C isolates for *Salmonella* Muenchen by multiplex PCR (*13*). We selected 13 heavy-breeder flocks that tested positive for *Salmonella* Muenchen on >2 successive collections as the positive group and 9 flocks with >3 successive negative results for *Salmonella* as the control group. We identified newly hatched broiler flocks (n = 78) and purposely sampled them for detection of *Salmonella* Muenchen: of those, 43 flocks were descendent from the positive group, and 35 flocks were descendent from the control group (Figure 1). We used those samples to estimate prevalence and assess risk factors for positivity to *Salmonella* Muenchen in broilers. In addition, 15–20 months after the initial broiler sampling, we resampled 10 new flocks in barns where *Salmonella* Muenchen was previously detected (hereafter, repeat broilers) (Figure 1). We obtained samples from broiler farms by using environmental drag swabs either inside the barn (interior swabs) or around the exterior of the barn (exterior swabs), or by using chick sampling and testing, conducted independently of this study (Appendix 2 Table 1). We conducted environmental sampling of the barns just before marketing, during marketing time (i.e., when transferred to the slaughterhouse at age 35–42 days), or after marketing when the barn was emptied but before cleaning.

We selected 70 of the *Salmonella* Muenchen isolates, representing a broad geographic distribution and representative of the different production stages, to undergo whole-genome sampling. That group included isolates from commercial broilers, subdivided into those descendent from the positive (n = 20) and control (n = 18) groups, hatcheries (n = 3), breeder flocks (n = 10), breeder pullets (n = 6), grandparent flocks (n = 3), and repeat broilers (n = 10) (Appendix 2 Table 3).

For analysis C, we chose 11 isolates that had undergone whole-genome sequencing and genetic analysis (from analysis A) to represent the different sources and genetic diversity of *Salmonella* Muenchen in Israel. In addition, we downloaded paired-end reads of *Salmonella* Muenchen global isolates (n = 125; i.e., strains collected during 2018–2021 from various sources in other countries) and strains known to contain pESI (*8*) from the National Center for Biotechnology Information (NCBI; https://www.ncbi.nlm.nih.gov) (Appendix 2 Table 4).

### Genetic Analysis

We generated paired-end reads for all isolates using the NextSeq platform (Illumina, https://www.illumina.com). In addition, we subjected isolate Br19 to long-read sequencing using the Oxford Nanopore platform (Nanopore Technologies, https://nanoporetech.). We used its hybrid assembly, built using Unicycler version 0.5.0 (github.com/rrwick/Unicycler) (*14*), as a genetically relevant reference genome. We aligned Illumina reads to the reference genome using Snippy version 4.6.0 (https://github.com/tseemann/snippy). We created maximum-likelihood trees using RAxML version 8.2.12 (https://github.com/amkozlov/raxml-ng) (*15*). We assessed branch support for the trees using 10,000 bootstrap replicates. We conducted de novo genome assemblies using SPAdes version 3.12.0 (*16*). We used contigs as an input for detection of AMR genes, known point mutations, and genes associated with virulence and resistance; detection of plasmid replicons; detection of a pESI target sequence (*8*); in silico serotyping; and multilocus sequence typing.

For analyses A and B, we rooted the maximum-likelihood trees to a *Salmonella* Muenchen sequence type (ST)112 outgroup strain (NCBI Sequence Read Archive accession no. SRR6222324). For analysis C, we rooted the tree to a *Salmonella* Braenderup strain (accession no. SRR5874793), a closely related yet genetically distinct serotype (*17*).

### Phenotypic Analysis

We assessed antimicrobial resistance of a selection of broiler isolates (n = 15), representing the diversity of genetic elements present that might confer resistance, using Sensititre susceptibility plates (Thermo Fisher Scientific, https://www.thermofisher.com). We used human clinical breakpoints for MIC value interpretation.

### Criteria for the Presence of the pESI Plasmid

We determined that the pESI plasmid was present if the plasmid replicon IncFIB (pN55391) or hyp_pESI, a 559bp target sequence (*8*), were present, together with >1 of 3 groups of genes previously described in pESI (*8*,*18*). Those gene groups included 4 AMR genes, *sul1*, *tetA*, *aadA1*, and *qacEdelta1*; yersiniabactin genes *ybtP* and *ybtQ*; or genes associated with the mer operon, *merC*, *merP*, *merT,* and *merR*.

### Statistical Analysis

For statistical analysis, we used WINPEPI version 11.65 (*19*). We calculated Pearson χ^2^, Fisher exact test, and odds ratios (ORs) with 95% CIs, as appropriate.

### Ethics Statement

Isolates used for the purpose of this research were collected as part of mandatory routine surveillance programs that were conducted according to the ethical standards and in compliance with Israel law. Clinical isolates from humans were obtained anonymously from the Ministry of Health, Public Health Laboratories, Jerusalem, Israel. Additional sampling was limited to environmental sampling of poultry farms.

## Results

### Analysis A—Genetic Variability of *Salmonella* Muenchen in Poultry and Humans

**Figure 2:**
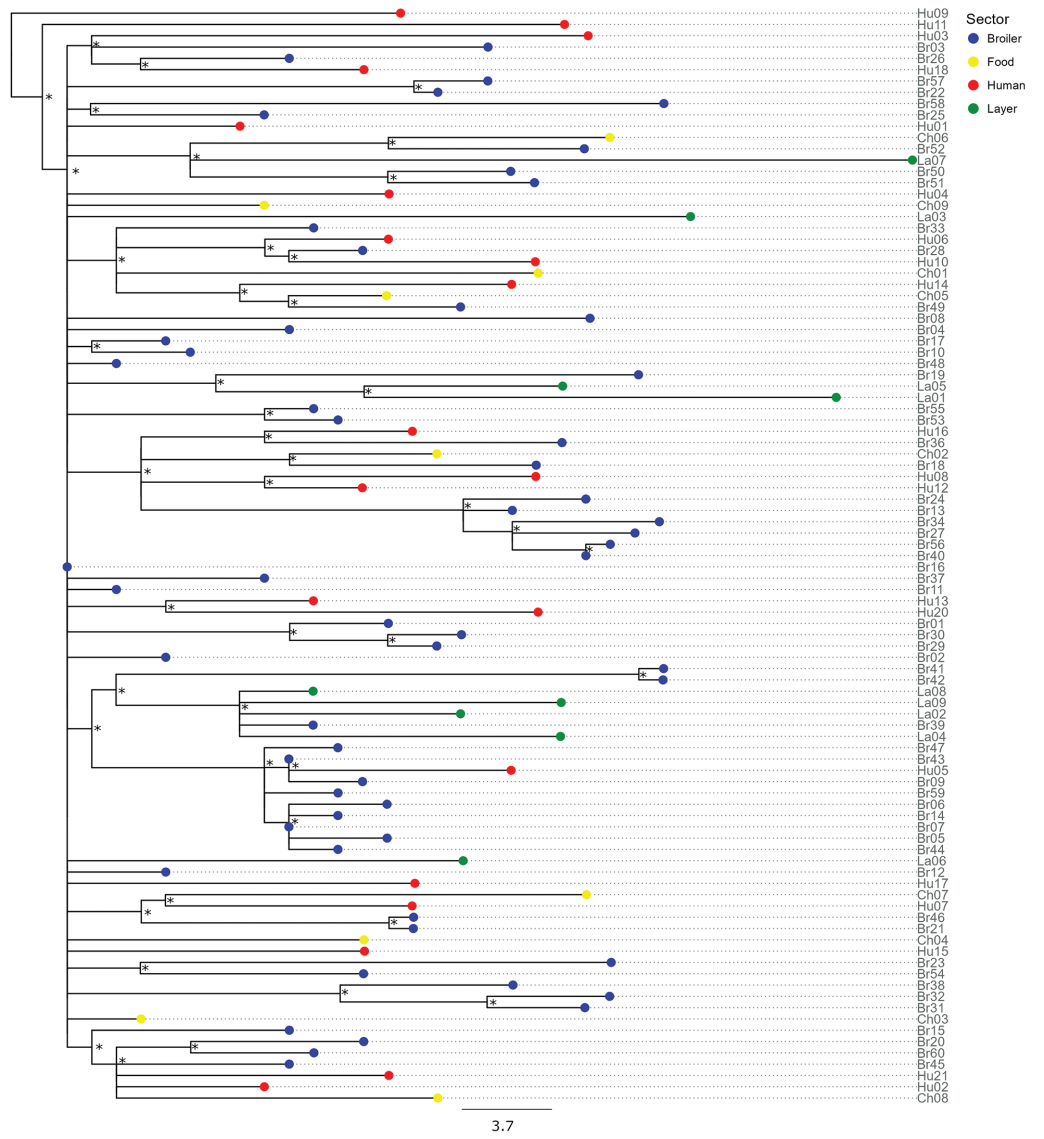
Maximum-likelihood tree reconstructed using whole-genome sequences of *Salmonella enterica* serovar Muenchen isolates collected from broilers (n = 60), layers (n = 9), food products of poultry origin (whole chicken carcasses sampled in poultry slaughterhouses; n = 9), and humans (n = 21) in study of *Salmonella* Muenchen in poultry and humans, Israel, June 2020–January 2022. We used a hybrid assembly of *Salmonella* Muenchen sequence type 82 (Br19) as a reference genome and used *Salmonella* Muenchen sequence type 112 (National Center for Biotechnology Information Sequence Read Archive accession no. SRR6222324) to root the tree (not shown). The analysis included 1,227 single-nucleotide polymorphisms (SNPs) in the alignment (0–69 pairwise SNPs, median 31). Because isolates Hu19 and Br35 were duplicates of isolate Br16, we removed them from the analysis. Tip colors indicate sector. Asterisks (*) indicate nodes with >70% bootstrap support. Additional data (Appendix 1 Figure 2) include a heatmap and show the presence of acquired antimicrobial resistance genes and point mutations and indicate the isolates in which antimicrobial sensitivity was tested in vitro and the presence of a pESI genetic marker, hyp_pESI, and plasmid replicons. Scale bar represents SNP difference.

We identified *Salmonella* Muenchen from all sources (n = 99) as ST82. Clustering according to source was not evident in the reconstructed maximum-likelihood tree (Figure 2). We identified 19 acquired AMR genes. We found the combination of *aadA1*, *sul1*, *tetA*, and *qacE* genes, associated with pESI and conferring resistance to streptomycin, sulfonamides, tetracyclines, and quaternary ammonium, in 92/99 (92.9%) isolates. We found that 86/99 (86.9%) isolates harbored the *dfrA14* gene, conferring resistance to trimethoprim, and 16/99 (16.2%) isolates harbored the *bla_T_*_EM_ gene, conferring resistance to β-lactams. In addition, 1 isolate from a chicken carcass harbored the *bla*_OXA-808_ gene. Genetic determinants associated with quinolone resistance were present in 58/99 (58.6%) isolates. We found the *qnrB19* gene, associated with the presence of the Col (pHAD28) plasmid replicon, in 40/99 (40.4%) isolates, and the *qnrS1* gene in 13/99 (13.1%) isolates. We found the S83A or S83F mutation in the DNA gyrase A (*gyrA)* gene in 6.1% (6/99) of isolates (Appendix 1 Figure 2; Appendix 2 Table 5). We further verified our findings using the Br19 hybrid assembly (Appendix 1). We found no significant difference in the presence of AMR genes across the different sources (Table 1). We identified the plasmid replicon IncFIB (pN55391), hyp_pESI, and the yersiniabactin genes *ybtP* and *ybtQ*, indicating the presence of pESI plasmid, in all but 1 strain, a human clinical isolate (Appendix 2 Table 5).

**Table 1:** Acquired antimicrobial resistance genes detected in strains of *Salmonella enterica* serovar Muenchen, by source, in study of *Salmonella* Muenchen in poultry and humans, Israel, 2020–2023

Source	Total	No. (%) isolates with resistance genes							
		Aminoglycoside	β-lactam	Florfenicol	Sulfonamide	Tetracycline	Trimethoprim	Quinolone	Quaternary disinfectant
Poultry									
Broiler	60	58 (96.7)	12 (20)	7 (11.7)	58 (96.7)	59 (98.3)	54 (90)	35 (58.3)	58 (96.7)
Food	9	9 (100)	2 (22.2)	2 (22.2)	8 (88.9)	9 (100)	8 (88.9)	5 (55.6)	8 (88.9)
Layer	9	9 (100)	0 (0)	0 (0)	9 (100)	9 (100)	9 (100)	3 (33.3)	9 (100)
Human	21	18 (85.7)	3 (14.3)	2 (9.5)	18 (85.7)	19 (90.5)	15 (71.4)	10 (47.6)	18 (85.7)
Total	99	94 (94.9)	17 (17.2)	11 (11.1)	93 (93.9)	96 (97)	86 (86.9)	53 (53.5)	93 (93.9)

Phenotypic analysis of broiler isolates (n = 15) showed that all were resistant to tetracycline and sulfisoxazole (Appendix 2 Table 2). Despite a few minor exceptions, we observed an overall alignment between phenotype and the genotype (Table 2). The presence of either the *qnrB19* or *qnrS1* gene or *gyrA* S83F mutation alone conferred decreased susceptibility to ciprofloxacin (MICs >0.12 and <1 µg/mL) in 9/11 (81.8%) and clinical resistance (MIC = 1 µg/mL) in 1/11 of the tested isolates. The *gyrA* S83F mutation was associated with resistance to nalidixic acid; the presence of the *gyrA* mutation together with the *qnr*S1 gene (n = 1) conferred clinical resistance to ciprofloxacin (MIC = 1 µg/mL).

**Table 2:** Phenotypic resistance and presence of acquired antimicrobial resistance genes known to confer resistance in *Salmonella enterica* serovar Muenchen in study of *Salmonella* Muenchen in poultry and humans, Israel, 2020–2023*

Antimicrobial class and drug	MIC cutoff, µg/mL	No. infections/no. tested (%)	Relevant AMR genes and point mutations present in tested isolates (no.)
Tetracyclines			
Tetracycline	16	15/15 (100)†	*tetA* (*13*), *tetA* + *tetM* (*1*)
Aminoglycosides			
Streptomycin	32	14/15 (93.3)	*aadA1* (*13*), *aadA1* + *aadA2* (*1*)
Gentamicin	16	0/15 (0)	None
Phenicols			
Chloramphenicol	32	6/15 (40.0)†	*floR* (*4*), *floR* + *cmlA1* (*1*)
Penicillins			
Ampicillin	32	7/15 (46.7)†	*bla*_TEM-1_ (5), *bla*_TEM-176_ (1)
Amoxicillin/clavulanic acid, 2:1 ratio	32/16	0/15 (0)	None
Cephalosporins			
Cefoxitin	32	0/15 (0)	None
Ceftriaxone	4	0/15 (0)	None
Folate pathway antagonists			
Sulfisoxazole	256	15/15† (100)	*sul1* (13), *sul2* (1)
Trimethoprim/sulfamethoxazole	4/76	14/15† (93.3)	*dfrA14 + sul1* (10), *dfrA14* (1), *dfrA12* + *dfrA14* + *sul2* (1)
Quinolones			
Ciprofloxacin	0.12‡	11/15 (73.3)	*qnrB19* (7§), *qnrS1* (3), *gyrA* S83F (1), *qnrS1* + *gyrA* S83F (1)
Nalidixic acid	32	4/15 (26.7)	*qnrB19* (1), *qnrS1* (1), *gyrA* S83F (1), *qnrS1* + *gyrA* S83F (1)
Macrolides			
Azithromycin	32	0/15 (0)	None
Carbapenems			
Meropenem	4	0/15 (0)	None

*AMR, antimicrobial resistance; +, genes found simultaneously on the same isolate. †>1 isolates exhibited phenotypic resistance without the identification of a relevant AMR gene ‡MIC cutoff for decreased susceptibility as a marker for emerging fluoroquinolone resistance (*20*). §One isolate (Br04) harbored the *qnrB19* gene but was sensitive to ciprofloxacin (MIC = 0.03 µg/mL)

### Analysis B—*Salmonella* Muenchen Transmission in the Broiler Production Industry

We determined *Salmonella* Muenchen prevalence of 64.0% (412/644) in group C samples collected as part of the active surveillance program from heavy-breeder flocks in 2021. We estimated *Salmonella* Muenchen prevalence in broiler flocks at 61.5% (95% CI 51.5%–71.5%; 48/78 isolates). We found no significant difference (p = 0.83) in prevalence between the broilers descendent from positive (26/43) and control (22/35) breeding groups. Moreover, we recovered *Salmonella* Muenchen isolates in 8/10 (80.0%) of the broiler barns in which it was previously detected, 15–20 months after the initial sampling; we referred to the poultry in those barns as repeat broilers.

We found no significant association between positivity in the breeder farm and detection of *Salmonella* Muenchen in chick samples (Table 3) or environmental swab samples collected in the barn (Table 4) and around the barn (Table 5). Positivity in the chicks was significantly associated with older age of chicks (OR = 8.1; p < 0.05). Positivity in the interior swab samples was associated with positivity in the exterior swabs collected at the same time (OR = 123; p < 0.01). In addition, positivity in the exterior swab samples was associated with sampling time, sampling after versus before marketing (OR = 8.25; p < 0.05).

**Table 3:** Risk factors for positivity to *Salmonella enterica* serovar Muenchen in broiler flocks by chick sampling, in study of *Salmonella* Muenchen in poultry and humans, Israel, 2020–2023

Risk factor	No. positive/no. tested (%)		Odds ratio (95% CI)	p value*
	First risk group	Second risk group		
Breeder group, positive vs. control	9/42 (21.4)	9/35 (25.7)	0.79 (0.24–2.61)	0.658
Integration member,† yes vs. no	10/52 (19.2)	8/25 (32)	0.51 (0.15–1.76)	0.215
Chick age, >2 weeks vs. <2 weeks	4/6 (66.7)	14 /71 (19.7)	8.14 (1.35–49.03)	0.024
Hatchery, positive or mixed vs. negative	14/63 (22.2)	4/14 (28.6)	0.71 (0.17–3.62)	0.728

*Calculated using Pearson χ^2^ test; if values to compare were <5, Fisher exact test was used. A significance level of α = 0.05 was applied. †Broiler farms contracted by a company that owns breeder farms, hatcheries, feed mills, and transportation.

**Table 4:** Risk factors for positivity to *Salmonella enterica* serovar Muenchen in broiler flocks by drag swab sampling taken from inside broiler barns in study of *Salmonella* Muenchen in poultry and humans, Israel, 2020–2023

Risk factor	No. positive/no. tested (%)		Odds ratio (95% CI)	p value*
	First risk group	Second risk group		
Breeder group, positive vs. control	18/25 (72)	18/24 (75)	0.86 (0.20–3.67)	0.812
Integration member, yes vs. no	26/36 (72.2)	9/12 (75)	0.87 (0.13–4.53)	1
Time of sampling				
During evacuation vs. before	17/23 (73.9)	8/10 (80)	0.71 (0.06–5.31)	1
After evacuation vs. before	11/16 (68.8)	8/10 (80)	0.55 (0.04–4.64)	0.668
Exterior swab, positive vs. negative	20/20 (100)	6/25 (24)	∞ (10.45–∞)	<0.001

*Calculated using Pearson χ^2^ test; if values to compare were <5, Fisher exact test was used. A significance level of α = 0.05 was applied.

**Table 5:** Risk factors for positivity to *Salmonella enterica* serovar Muenchen in broiler flocks by drag swab sampling taken from the exterior environment of broiler barns in study of *Salmonella* Muenchen in poultry and humans, Israel, 2020–2023

Risk factor	No. positive/no. tested (%		Odds ratio (95% CI)	p value*	
	First risk group	Second risk group)			
Breeder group, positive vs. control	14/25 (56)	12/21 (57.1)	0.95 (0.25–3.58)	0.938
Integration member, yes vs. no	18/35 (51.4)	8/11 (72.7)	0.40 (0.06–2.06)	0.302
Time of sampling				
During evacuation vs. before	12/22 (54.6)	2/8 (25)	3.60 (0.47–42.40)	0.226
After evacuation vs. before	11/15 (73.3)	2/8 (25)	8.25 (0.87–105.55)	0.039
Interior swab, positive vs. negative	20/26 (76.9)	0/19 (0)	∞ (10.45–∞)	<0.001

*Calculated using Pearson χ^2^ test; if values to compare were <5, Fisher exact test was used. A significance level of α = 0.05 was applied.

**Figure 3:**
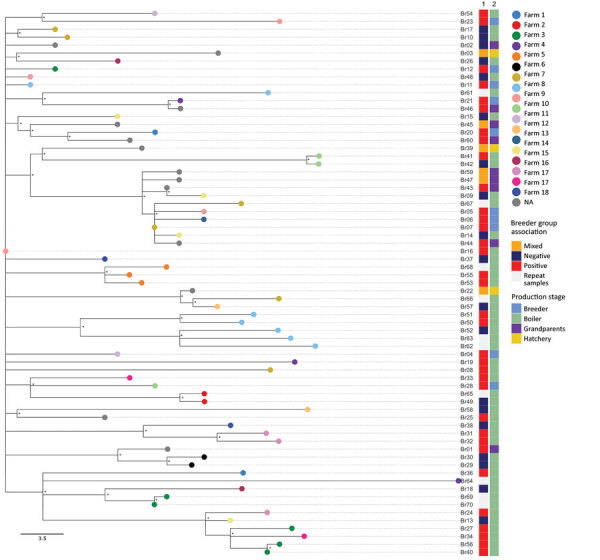
Maximum-likelihood tree reconstructed using whole-genome sequences of *Salmonella enterica* serovar Muenchen isolates collected from the various stages of the broiler production industry in study of *Salmonella* Muenchen in poultry and humans, Israel, June 2020–June 2023. We used a hybrid assembly of *Salmonella* Muenchen sequence type 82 (Br19) strain as a reference genome and used *Salmonella* Muenchen sequence type112 (National Center for Biotechnology Information Sequence Read Archive accession no. SRR6222324) to root the tree (not shown). The analysis included 874 single-nucleotide polymorphisms (SNPs) in the alignment (0–61 pairwise SNPs, median 30). Tip colors indicate the commercial broiler farm either where barns were tested (in the case of broiler samples), or the commercial broiler farm to which the breeder flock supplied (breeder samples). Asterisks (*) indicate nodes with >70% bootstrap support. Column 1 indicates data for the breeder group to which the broiler flocks were related (positive or control/negative). We labeled hatcheries and grandparent flocks as mixed if they received/supplied chicks from/to both positive and control heavy breeder farms. Column 2 indicates data for the production stage from which the sample was collected. Scale bar represents SNP difference.

Clustering of strains collected from parent breeding flocks and the descendant broiler flocks was not evident in the reconstructed phylogeny tree (Figure 3). In addition, we observed no genetic similarity between grandparents, pullets, hatcheries, and the breeder flocks, except for an instance of an isolate from a pullet flock (Br46) persisting (difference of 2 single-nucleotide polymorphisms [SNPs]) in the same flock when we sampled it 10 months later as a heavy-breeder flock (Br21). We observed evidence of clustering of samples collected by various sampling techniques (chicks, interior swabs, and exterior swabs) from the same barn in 7/13 barns (Figure 3). Repeat sampling of the broiler barns produced isolates that were closely related in 6/10 (60.0%) of the cases; 4 of those were highly genetically similar (4–8 SNPs) to the previous isolates from the same farm (Figure 3).

Although the samples we collected in various production stages were genetically heterogeneous, we found the frequencies of AMR gene presence in different production stages to be similar, based on a limited number of samples compared between sources (Table 6). We found similar frequencies of AMR genes in the positive and control groups (p>0.05) Table 7).

**Table 6:** Acquired antimicrobial resistance genes detected in strains of *Salmonella enterica* serovar Muenchen collected in the broiler industry, by production stage, in study of *Salmonella* Muenchen in poultry and humans, Israel, 2020–2023

Production stage	No.	No. (%) isolates with resistance genes							
		Aminoglycoside	β-lactam	Florfenicol	Sulfonamide	Tetracycline	Trimethoprim	Quinolones	Quaternary disinfectant
Grandparentand pullets	9	8 (88.9)	1 (11.1)	0	8 (88.9)	9 (100)	8 (88.9)	3 (33.3)	8 (88.9)
Breeder	10	10 (100)	1 (10)	0	10 (100)	10 (100)	8 (80)	6 (60)	10 (100)
Hatchery	3	3 (100)	0	0	3 (100)	3 (100)	3 (100)	0	3 (100)
Broiler	38	37 (97.4)	10 (26.3)	7 (18.4)	37 (97.4)	37 (97.4)	35 (92.1)	26 (68.4)	37 (97.4)
Broiler repeat	10	9 (90)	3 (30)	3 (30)	9 (90)	10 (100)	10 (100)	7 (70)	9 (90)
Total	70	67 (95.7)	15 (21.4)	10 (14.3)	67 (95.7)	69 (98.6)	64 (91.4)	42 (60)	67 (95.7)

**Table 7:** Acquired antimicrobial resistance genes detected in strains of *Salmonella enterica* serovar Muenchen collected in broiler flocks, by parent breeder flock status, in study of *Salmonella* Muenchen in poultry and humans, Israel, 2020–2023

Broiler group	No.	No. (%) isolates with resistance genes							
		Aminoglycoside	β-lactam	Florfenicol	Sulfonamide	Tetracycline	Trimethoprim	Quinolones	Quaternary disinfectant
Positive	20	19 (95)	8 (40)	4 (20)	19 (95)	19 (95)	19 (95)	16 (80)	19 (95)
Control	18	18 (100)	2 (11.1)	3 (16.7)	18 (100)	18 (100)	16 (88.9)	10 (55.6)	18 (100)
Total	38	37 (97.4)	10 (26.3)	7 (18.4)	37 (97.4)	37 (97.4)	35 (92.1)	26 (68.4)	37 (97.4)

### Analysis C—Global Distribution of *Salmonella* Muenchen Harboring pESI

We reconstructed a maximum-likelihood phylogenetic tree from 11 isolates chosen from our study and 125 isolates from elsewhere globally (Appendix 1 Figure 3). We noted that *Salmonella* Muenchen was genetically diverse globally, represented by 11 different STs in our analysis. We distinguished 2 main genetic subpopulations; 1 genetic subpopulation consisted of ST83 (n = 38), and the other contained predominantly ST112 (n = 25) and ST82 (n = 49). ST83 was detected in the United States, Canada, and the United Kingdom from both human and food-animal sources. ST112 had a broader distribution, including strains from the United States, Canada, Mexico, United Kingdom, and Australia. We identified pESI exclusively in ST82 isolates. ST82, which also included isolates without pESI, was found in most of the countries represented. The pESI-positive ST82 isolates formed a clade and included all the isolates from our study and an additional 18 strains from other countries including the United States (n = 12), United Kingdom (n = 5), and South Africa (n = 1). Apart from the isolates collected in Israel representing both human and poultry sources, pESI-positive isolates were from human samples (n = 15) or from isolates from the NCBI database that lacked detailed information about source (n = 3) (Appendix 2 Table 4,6).

## Discussion

*Salmonella* Muenchen has become the dominant serovar in Israel; a surge in human clinical cases was associated with an increased prevalence in the poultry industry (*7*). We demonstrated genetic similarity, including the presence of the pESI megaplasmid, in isolates collected from poultry and chicken meat and from humans. Furthermore, we estimated a high prevalence (61.5% [95% CI 51.5%–71.5%]) of the emerging *Salmonella* Muenchen in broilers, and by tracing transmission pathways within the broiler industry we demonstrated that horizontal transmission plays a major role in its spread. Finally, we found that the emerging multidrug-resistant *Salmonella* Muenchen strain in Israel closely resembles pESI-positive *Salmonella* Muenchen isolates from other countries, underscoring the potential for clonal expansion and the risk to public health.

Various sources, such as food (*21*) and animal hosts (*22*), are associated with NTS infections, and circulation between animal hosts, which could potentially form additional transmission pathways to humans, have been described previously (*23*). *Salmonella* Muenchen has been detected in a wide range of foods of animal origin, including beef, seafood, and pork (*24*), and in plant-based foods (*25*). Chicken meat has rarely been documented as a source of infection; the last reported outbreak in the United States occurred in 2015 (*26*). However, our findings strongly suggest that poultry broilers are an important source of human infections caused by *Salmonella* Muenchen in Israel. The high genetic similarity between strains collected from broiler flocks and layer flocks may indicate that eggs serve as an additional source for human infections, however their role as a route of transmission to humans needs to be further explored.

Controlling *Salmonella* Muenchen in the poultry industry in Israel will be crucial to curb the incidence of infections in the human population. Vaccinating breeder flocks can be effective (*2*), assuming that the transmission of the pathogen is mainly vertical from the parent to the production flocks. A previous study (*27*) examining the transmission of *Salmonella* within vertically integrated poultry operations suggested, on the basis of serologic and molecular methods, that transmission pathways are varied and might depend on the serotype; certain serotypes, including *Salmonella* Muenchen, transmitted horizontally to broiler flocks after hatching. Our findings, supported by advanced large-scale genetic analysis, further support that route of transmission in the emerging *Salmonella* Muenchen spread. Our conclusion is based on the lack of clustering of isolates from broiler flocks with their parent breeding flocks and lack of significant risk for *Salmonella* Muenchen positivity in broiler flocks descending from *Salmonella* Muenchen–positive breeder flocks. Additional findings supported on-site infection and horizontal transmission, such as the significantly increased risk for *Salmonella* Muenchen positivity in older chicks, genetic clustering of isolates within and between broiler farms, and evidence of strains persisting in broiler barns over time. Therefore, we suspect that the initiation of mandatory vaccination of breeder flocks in Israel against *Salmonella* Muenchen as of March 2024 (Bulletin notice, Ministry of Agriculture, 2023 Aug) may result in a limited reduction in the prevalence of *Salmonella* Muenchen, because *Salmonella* persists in the farm soil or barn litter (*28*). Other possible measures that can be employed on the farms or along the production chain (e.g., during transport) to decrease the prevalence of *Salmonella* Muenchen include increasing biosecurity by different measures, such as controlled farm access and thorough cleaning and disinfection between flocks. Sources for *Salmonella* contamination, including poultry feed and pests (*29*–*31*), may also be controlled. In addition, techniques such as fecal microbiota transplants to chicks to prevent colonization of *Salmonella* spp. via competitive exclusion and the use of probiotics are potential methods to reduce gastrointestinal colonization (*31*,*32*). Increased surveillance and control of this serotype along the production chain, such as during transport, in the slaughterhouse, and during processing, might also reduce transmission to the human consumer.

The public health risk associated with transmission of multidrug-resistant *Salmonella* is considered one of the biggest threats to global health (*33*). Surveillance data from the Ministry of Health reported that 30/77 (39.0%) of *Salmonella* Muenchen isolates collected in 2021 from humans and 21/41 (51.2%) from nonhuman sources had reduced susceptibility to ciprofloxacin (*7*), which is critical for treatment of invasive salmonellosis in humans (*34*). In our study, 11/21 (52.4%) of human and 47/78 (60.3%) of poultry isolates contained chromosomal point mutations on the *gyrA* gene, or harbored *qnr* genes known to be involved in conferring resistance to quinolones (*35*). Phenotypic testing revealed that all isolates exhibiting decreased susceptibility or resistance to ciprofloxacin had >1 of those resistance determinants present. The presence of the transmissible *bla*_OXA-808_ gene in an isolate recovered from poultry meat was unusual. That gene, belonging to the *bla*_OXA-23_ family, a group of carbapenem-hydrolyzing β-lactamases, was previously reported in *Acinetobacter* species (*36*). Although *Salmonella* has been known to harbor *bla*_OXA_ genes (*37*), this specific variant had not been previously reported. Potential carbapenem resistance is an alarming finding because carbapenem is a broad-spectrum antimicrobial drug reserved as a last-resort treatment for bacterial infections in humans (*34*).

Phenotypic resistance aligned with genotype with a few exceptions: tetracycline, chloramphenicol, ampicillin, sulfisoxazole, and trimethoprim/sulfamethoxazole. We observed 3 MDR isolates exhibiting resistance to >1 of those antimicrobial drugs with no known AMR gene present. Such discordances may be the result of various potential mechanisms, such as AMR determinants yet to be described (*38*).

The surge in prevalence of *Salmonella* Muenchen in poultry since 2018 correlated with a marked increase in the frequency of pESI-positive isolates: from 146 *Salmonella* Muenchen isolates collected by the EPB during 2009–2021, pESI was detected in only 3/50 (6.0%) of isolates before 2018 but in 85/96 (88.5%) of isolates since January 2018 (E. Elnekave, unpub. data). In this study, we demonstrated the continuation of the trend; 100.0% (88/88) of poultry isolates harbored the plasmid. The pESI plasmid provides fitness advantages to the bacterial host, such as increased tolerance to mercury and oxidative stress, and AMR (*9*). Those advantages might contribute to the high prevalence and spread of the pESI-harboring *Salmonella* Muenchen; further studies are required to determine which of the specific advantages and under what conditions enable *Salmonella* Muenchen propagation in the broiler industry in Israel.

We found that the Israel *Salmonella* Muenchen strains were genetically similar to strains collected in the United Kingdom, South Africa, and the United States. All belonged to ST82 and harbored pESI. Such clonality might suggest travel-associated transmission of *Salmonella* Muenchen or might be a result of a common origin and global dissemination of contaminated poultry breeding stock, as suggested previously with this serotype (*8*) and others (*39*). In addition, the strain was recently reported from the West Bank (*40*), which suggests regional spread. The relatively rare identification of pESI in strains other than *Salmonella* Infantis ST32 and *Salmonella* Muenchen ST82 (*18*) might indicate an adaptation mechanism to the plasmid that has not been determined. Given the vast global spread of *Salmonella* Infantis harboring pESI (*11*) since its detection (*9*), it is highly possible that a pESI-harboring *Salmonella* Muenchen ST82 will be the next *Salmonella* to emerge globally.

Appendix 1 Additional information about multidrug-resistant pESI-harboring *Salmonella enterica* serovar Muenchen sequence type 82 in poultry and humans, Israel, 2020–2023.

Appendix 2 Data from study of multidrug-resistant pESI-harboring *Salmonella enterica* serovar Muenchen sequence type 82 in poultry and humans, Israel, 2020–2023.
