# Supplementary material for: Multidrug-Resistant pESI-Harboring Salmonella enterica Serovar Muenchen Sequence Type 82 in Poultry and Humans, Israel, 2020–2023
Source: Emerg Infect Dis. 2025 Oct;31(10):1969–79. doi: 10.3201/eid3110.250191 (PMC12483013; doi:10.3201/eid3110.250191)
Supplement: Appendix 1 — Additional information about multidrug-resistant pESI-harboring Salmonella enterica serovar Muenchen sequence type 82 in poultry and humans, Israel, 2020–2023. [file 25-0191-Techapp-s1.pdf]

Article DOI: https://doi.org/10.3201/eid3110.250191

EID cannot ensure accessibility for supplementary materials supplied by authors. Readers who have difficulty accessing supplementary content should contact the authors for assistance.

Multidrug-Resistant pESI-Harboring *Salmonella enterica* Serovar Muenchen Sequence Type 82 in Poultry and Humans, Israel, 2020–2023

Appendix

Materials and Methods

Study Populations

Determining the genetic variability of *S.* Muenchen across poultry sectors and human clinical cases in Israel (Analysis A)

Salmonella Muenchen isolates from the broiler production industry, layer hens, poultry slaughterhouses and human clinical cases (n = 99) were collected in Israel and were subjected to whole genome sequencing (WGS). The isolates were chosen to represent their sector with a broad geographic distribution (Appendix 1 Figure 1). Isolates from the broiler production sector (n = 60) were obtained as part of the isolate collection for analysis B (see below) between June 2020 and January 2022. Isolates from layers flocks (n = 9) were recovered as part of the national surveillance program by the Egg and Poultry Board (EPB) and the Israeli Veterinary Services in 2021. Isolates from food sources (n = 9) were recovered from whole chicken carcasses in poultry slaughterhouses in 2021 as part of a national surveillance for food safety and were provided by the microbiology food safety laboratory at the Kimron Veterinary Institute. Isolates (n = 21) that were collected from human clinical cases in 2021 were selected from the frozen (−80°C) isolate collection of the national reference laboratory of the Ministry of Health (MOH). These isolates were provided without patient data; however, they were chosen by the MOH collaborators to represent a broad geographic distribution.

Tracing *S*. Muenchen Transmission in the Broiler Production Industry (Analysis B)

Salmonella Muenchen isolates collected from broilers (n = 48; further subdivided into those descendent from the Positive group (n = 20), Control group (n = 18) and repeat barns (n = 10); see below), hatcheries (n = 3), breeder flocks (n = 10), breeder pullets (n = 6) and grandparent flocks (n = 3), were selected to be included in this analysis.

Global Distribution of *S*. Muenchen Harboring pESI (Analysis C)

Isolates collected from Israel (n = 11, including human (n = 3), broilers (n = 5), a layer (n = 1) and food (n = 2)) were chosen from the phylogenetic tree created in analysis A (see above) to represent the diversity of *S*. Muenchen found in Israel. “Global isolates” were *S*. Muenchen strains (n = 125) from other countries that were selected from the 'pathogen detection' database at the National Center for Biotechnology Information (NCBI) (https://www.ncbi.nlm.nih.gov/pathogens/) according to the criteria: Illumina sequences of *Salmonella* serotypes Muenchen or Virginia collected between 2018 and 2021 from human, animal, food or environmental sources. From each location, ten samples from each year were randomly selected, or all available samples were taken if sample number was less than ten. In addition, we included various *S*. Muenchen strains known to contain pESI that were previously described in Cohen et al. (*1*). Overall, 140 sequences were downloaded from NCBI and considered for inclusion in our study.

Sample Collection

Heavy breeders, grandparent and hatchery sampling, *Salmonella* isolation and detection of *S*. Muenchen: Sampling of heavy breeder, grandparent flocks and hatcheries was carried out as part of the National *Salmonella* active Surveillance program between June 2020 and January 2022. Environmental samples were collected from the barns and hatcheries using *Salmonella* Drag Swabs (Hylabs, Rehovot, Israel). The samples were processed by the *Salmonella* diagnostic laboratory, EPB, following International Standards Organization (ISO) protocol ISO-6579 −1, Amendment 1:2007 (*2*). Briefly, swabs were transferred to sterile containers filled with Buffered Peptone Water (BPW) (Hylabs, Rehovot, Israel) and incubated for 24 hours at 37°C. One hundred µl from each sample was applied in three drops to a Modified Semisolid Rappaport-Vassiliadis (MSRV) plate (Sigma-aldrich, Maryland, USA) and incubated at 41.5°C for 24 hours or 48 hours if insufficient growth was observed after the initial 24 hours. Suspect colonies were streaked onto Xylose Lysine Deoxycholate (XLD) and Brilliant Green

(BG) plate (Divided petri dish, Hylabs, Rehovot, Israel) and inoculated into a Hy-Enterotest® (Hylabs, Rehovot, Israel), to confirm the presence of *Salmonella*. Sero-grouping was carried out using the Slide Agglutination method using appropriate commercial antibodies (Becton Dickinson Company, USA; Staten Serum Institute, Denmark). For the purpose of this study, all *Salmonella* Group C isolates (those that agglutinated with specific O antisera for group C (O: 6,7,8)) were brought to the laboratory of Veterinary Public Health at the Koret Veterinary School on Luria Bertani (LB) agar plates to be screened for the presence of *S*. Muenchen using a multiplex PCR-assay (*3*).

Breeder and descendent broiler cohort selection: Thirteen heavy breeder flocks where *S*. Muenchen had been detected on at least two successive collections between May and July 2021 were selected as the Positive group. Nine heavy breeder flocks that had at least three successive negative results to *Salmonella* between May and July 2021 were selected to be the Control group (Appendix 2 Table 1). Newly hatched broiler flocks, descendent from the Positive and Control heavy breeder flocks and representing a wide geographic range were identified and sampled for detection of *S*. Muenchen (sample collection from those flocks is described below). Overall, 78 broiler flocks were tested: 43 with flocks descendent from the Positive group, and 35 with flocks descendent from the Control group. This sample size was sufficient to estimate the national prevalence of *S*. Muenchen in broiler flocks (n = 750) - the minimal sample size required for estimating prevalence with a 95% confidence that the result will be no more than 10% from the true prevalence, given an estimated prevalence of 30% is 77 flocks (*4*–*6*).

Repeat Broilers: Fifteen to twenty months after the initial broiler sampling, ten of the barns where *S*. Muenchen was previously detected, and which continued to house broiler flocks were resampled (hereafter: "repeat broilers"). These 10 barns were chosen to represent the geographic distribution and included farms with multiple broiler barns. These barns had undergone several periods where the broiler flocks had been marketed (i.e., sent to the slaughterhouse at the end of the growing period – ranging between 35 and 42 days of age), the barn cleaned and restocked with new flocks.

Broiler sampling, *Salmonella* isolation, detection of *S*. Muenchen: Similar to the national surveillance program, samples were obtained by using either environmental drag swabs inside the barn (interior swabs) or around the exterior of the barn (exterior swabs), or by using

chick samples collected at the farm. For each barn that housed a broiler flock, at least one of these methods was used (Appendix 2 Table 1). For the Repeat broiler sampling, all barns were sampled with interior and exterior swabs only.

Chick sampling was conducted independently of this study as part of routine procedures carried out by attending veterinarians at the EPB for detection of *Salmonella*, according to protocol ISO 6579–1:2017(E) (*2*). For each new broiler flock, ten chicks up to the age of 2.5 weeks, were sent to the EPB diagnostic laboratories for postmortem examination and bacterial detection from the heart, spleen, liver and intestines.

Environmental sampling of the barns was carried out by the researcher (TR, JP) or the farmer using *Salmonella* Drag Swabs (Hylabs, Rehovot, Israel). Samples were processed for isolation of group C *Salmonella* at the EPB and brought to the laboratory of Veterinary Public Health, Koret veterinary school, for detection of *S*. Muenchen (as described above in "Heavy breeders, grandparent and hatchery sampling, *Salmonella* isolation and detection of *S*. Muenchen"). Seventy of the *S*. Muenchen samples isolated were subsequently selected to undergo WGS (as described in the "Materials and methods" section of the manuscript, Analysis B).

Whole genome sequencing: All isolates were cultivated on LB agar plates locally or brought plated to the Laboratory of Veterinary Public Health at the Koret Veterinary School, Rehovot. DNA was extracted using the DNeasy Blood & Tissue kit (QIAGEN, Venlo, Netherlands) and quantified with a NanoDrop 2000 Spectrophotometer (Thermo Fisher Scientific, USA). Samples were sent to the Center for Genomic Technologies at the Hebrew University (Jerusalem, Israel) or to SeqCenter (Pittsburgh, PA, USA) for sequencing. In both centers, library preparation was performed using the tagmentation-based Illumina DNA Prep kit (https://www.illumina.com/products/by-type/sequencing-kits/library-prep-kits/illumina-dna-prep.html). Paired-end reads of 150 bp were generated using the NextSeq platform (Illumina, San Diego, CA).

In addition, one isolate from the broiler samples (Br19), as representative of a core genome from our cohort of isolates, and a distinctive AMR profile, was selected to create a hybrid assembly to be used as a reference genome. For this isolate, in addition to the Illumina short-read sequencing described above, DNA was extracted using the Blood and Cell Culture

DNA Midi kit (QIAGEN, Venlo, Netherlands) and sent to SeqCenter, where long-read sequencing performed using the Oxford Nanopore platform (Oxford, UK) created 178,292 reads with mean read length of 4,203bp.

Genetic analysis: Illumina raw reads were trimmed and checked for quality control using FastP v0.23.4 (*7*). Snippy v4.6.0 (*8*) with default settings was used to align all sequences to the relevant reference genome (in each analysis). Phage regions within the reference genome were found using PHASTER (*9*,*10*) and masked using a previously described script (https://git.io/vSiBA (*11*)). Recombination regions were detected and masked using Gubbins v3.3.5 (*12*). The alignment consisted of 1,227 SNP sites (between 0 and 69 pairwise SNPs, median = 31). Maximum- likelihood trees were reconstructed using RAxML v8.2.12 (*13*) and rooted to a *S*. Muenchen ST112 outgroup strain (SRR6222324) for analyses A and B, and a *S*. Braenderup (SRR5874793) outgroup strain for analysis C. Branch support for the trees were assessed using 10,000 bootstrap replicates. The trees were visualized using FigTree v1.4.4 (*14*) and displayed using R packages: ggtree v3.6.2 (*15*), treeio v1.22.0 (*16*), tidytree v0.4.2 (*17*) and tidyverse v2.0.0 (*18*).

De-novo genome assemblies were conducted using 'SPAdes' de novo assembler v3.12.0 (*19*) with the "careful" option to reduce short indels and minimize the number of mismatches in the final assembly. The quality of the assemblies was used as a threshold for sequences inclusion in the final analyses: i) The assemblies N50 were evaluated using QUAST v5.2.0 (*20*), and assemblies with N50 lower than 30,000 nt were excluded from the analyses. ii) Genome indexing was performed using bowtie2 v2.4.5 (*21*,*22*) and samtools v16.1 (*23*) and the average coverage depth of each assembly was calculated using BBmap v39.06 (*24*). Only sequences with average coverage depth greater than 20 were included in the analyses.

In addition, contigs were used as an input for: (a) detection of antimicrobial resistance genes, point mutations, virulence and stress response genes using AMRFinderPlus v3.12.8 with database version 2024–01–31.1 (*25*) that were uploaded and run on a local server. Default settings for detection threshold of minimum 50% coverage and 90% identity were used; (b) PlasmidFinder v2.1.6 (*26*) with the database updated on 2024–03–07 was used locally for in silico detection and typing of plasmids with default detection settings of 60% coverage and 90% identity; (c) A Basic Local Alignment Search Tool (BLAST) (*27*) was used on a local server to

assess the presence of a 559bp target sequence as described by Cohen et al, 2022 (*1*), as a marker for the pESI plasmid (please refer to the manuscript for the detailed criteria used for pESI identification); (d) In-silico serotyping using the *Salmonella* In Silico Typing Resource (SISTR) platform v1.1.1 (*28*) and (e) MLST v2.23.0 (https://github.com/tseemann/mlst) was used for multilocus sequence typing using the PubMLST database (*29*).

A hybrid assembly of strain Br19 was built combining the Oxford Nanopore long-reads with short-reads from the Illumina sequencing using Unicycler v0.5.0 (*30*) and was visualized with Bandage v0.8.1 (*31*). The location of known antimicrobial resistant genes and plasmid sequences were identified using a built-in BLAST (in Bandage).

Phenotypic Antimicrobial Resistance: Antimicrobial resistance was assessed using Sensititre susceptibility plates suitable for non-fastidious gram-negative isolates (plate CMV4AGNF, Thermo Scientific, West Sussex, UK, 2019). Plates contained a panel of 14 antimicrobials (i.e., amoxicillin-clavulanic acid, ampicillin, azithromycin, cefoxitin, ceftriaxone, chloramphenicol, ciprofloxacin, gentamicin, meropenem, nalidixic acid, streptomycin, sulfamethoxazole-trimethoprim, sulfisoxazole, and tetracycline). Microdilution plates were manually inspected and interpreted according to the Clinical and Laboratory Standards Institute (CLSI) cutoffs for *Salmonella* strains described in the guidelines (*32*). Azithromycin has no CLSI breakpoint therefore the National Antimicrobial Resistance Monitoring System (NARMS) cutoffs were used (*33*). Human cutoffs were used for the data to be relevant to the public health risk.

Risk factors for positivity in the broiler population: Data regarding the barn, flock and sampling results were provided by the EPB. Membership in large poultry integration distinguished broiler farms that were contracted by a company that owns breeder farms, hatcheries, feed mills and transportation, from those that were not.

Data summary and statistical analysis: Data was arranged and analyzed using R studio v4.2.0 (*34*), Microsoft Excel was used for frequency tables, and WINPEPI v11.65 (*35*) was used for Pearson chi square and odds ratio (OR) and confidence intervals of 95% (CI95%) calculations. Location of samples was mapped using ArcGIS Pro 3.2.2 (ESRI, Redlands, CA, USA). Metadata and accession numbers from sequenced isolates and those downloaded from NCBI database can be found in Appendix 2 Table 4.

Results

i) Genetic Variability of *S*. Muenchen in Poultry and Humans (Analysis A)

Appendix 1 Figure 1 shows the geographic distribution of samples used in the study.

The identification of pESI plasmid in all *S*. Muenchen isolates, except one human isolate, and the association between the presence of *qnrB19* gene and Col (pHAD28) plasmid, were further verified using the 'Br19' hybrid assembly. This isolate was chosen for its unusual pattern of acquired AMR genes, with the aim to investigate the presence of plasmids and the location of these AMR genes. The assembly included a 4,856,828bp circular chromosome (CP175968) and six circular plasmids (size range 2,960 to 273,967bp). The largest plasmid (CP175969) was blasted against the pESI plasmid (285,167bp; CP088902.1) (*36*), with a query match of 96% coverage and 100% identity. This plasmid contained the *dfrA14* and *tetA* genes, however the *sul1* gene, commonly found in pESI, was not identified. The smallest plasmid contained the *qnrB19* gene and the Col (pHAD28) plasmid replicon (CP175974). *Sul2, tetM, dfrA12, floR, qacL, cmlA, aadA1* and *aadA2* genes were found on a 111,917bp IncFIB (AP001918) plasmid (CP175970). On phenotypic analysis of 15 broiler isolates, all were resistant to tetracycline and sulfisoxazole (detailed MIC values are presented in Appendix 2 Table 2). Overall, the resistance patterns aligned with those expected from the genotype (Table 2), with the following exceptions: i) One isolate (Br04) was sensitive to ciprofloxacin despite harboring the *qnrB19* gene, ii) An isolate (Br08) was resistant to tetracycline, streptomycin and sulfisoxazole although relevant AMR genes were not identified, iii) Br12 was resistant to sulfamethoxazole-trimethoprim, chloramphenicol and ampicillin without relevant genes being identified and iv) Br57 was resistant to sulfamethoxazole-trimethoprim without relevant resistance genes.

ii) *S*. Muenchen Transmission in the Broiler Production Industry (Analysis B)

Prevalence in the different production sectors - The *S.* Muenchen prevalence of 64.0% (412/644) found in samples collected as part of the *Salmonella* active surveillance program from heavy-breeder flocks in 2021 in Israel, represented 80.8% (412/510) of the *Salmonella* serogroup C isolates from the heavy-breeder flocks. *Salmonella* serogroup C isolates detected in the less frequent surveillance scheme from hatcheries and grandparents (stock and pullets) were tested for the presence of *S*. Muenchen with 9/18 (50%) and 77/102 (75.5%) testing positive respectively. Of the total *Salmonella* serogroup C isolated from commercial broiler farms, 80/98

(81.6%) from the original sampling, and a further 15/17 (88.2%) from repeat sampling, were identified as *S*. Muenchen.

On-farm transmission - Based on the phylogenetic tree (Figure 3), in barns where chicks and at least one other sample (interior or exterior swab) was sequenced, the isolate from the chick shared a clade with at least one of the swab isolates in 5/8 (62.5%) of the cases. Moreover, in cases where more than one barn on the same farm was sampled (five farms), the isolates from different barns were closely related in three farms. On two of these farms, isolates differed by two SNPs confirming direct transmission between barns.

References

1. Cohen E, Kriger O, Amit S, Davidovich M, Rahav G, Gal-Mor O. The emergence of a multidrug resistant *Salmonella* Muenchen in Israel is associated with horizontal acquisition of the epidemic pESI plasmid. Clin Microbiol Infect. 2022;28:1499 e7- e14.

2. International Organization for Standardization. ISO 65791:2017 + A1:2020. Microbiology of the food chain—horizontal method for the detection, enumeration and serotyping of Salmonella. Part 1: detection of *Salmonella* spp. Geneva: The Organization; 2017 [cited 2024 Oct 2]. https://www.iso.org/standard/56712.html

3. Arnold K, Lim S, Rakler T, Rovira A, Satuchne C, Yechezkel E, et al. Using genetic markers for detection and subtyping of the emerging *Salmonella enterica* subspecies enterica serotype Muenchen. Poult Sci. 2022;101:102181. PubMed https://doi.org/10.1016/j.psj.2022.102181

4. Champely S. Pwr: basic functions for power analysis. R package version 1.3–0 ed; 2020.

5. Zhang G. TrialSize: R Functions for Chapter 3,4,6,7,9,10,11,12,14,15 of sample size calculation in clinical research. R package version 1.4 ed; 2020.

6. Stevenson MSE, Firestone S. epiR: tools for the analysis of epidemiological data. R package version 2.0.66 ed; 2023.

7. Chen S. Ultrafast one-pass FASTQ data preprocessing, quality control, and deduplication using fastp. iMeta. 2023;2:e107. PubMed https://doi.org/10.1002/imt2.107

8. Seemann T. Snippy: fast bacterial variant calling from NGS reads [cited 2024 Oct 2]. https://githubcom/tseemann/snippy

9. Arndt D, Grant JR, Marcu A, Sajed T, Pon A, Liang Y, et al. PHASTER: a better, faster version of the PHAST phage search tool. Nucleic Acids Res. 2016;44(W1):W16-21. PubMed https://doi.org/10.1093/nar/gkw387

10. Zhou Y, Liang Y, Lynch KH, Dennis JJ, Wishart DS. PHAST: a fast phage search tool. Nucleic Acids Res. 2011;39:W347–52.

11. Elnekave E, Hong S, Mather AE, Boxrud D, Taylor AJ, Lappi V, et al. *Salmonella enterica* serotype 4,[5],12:i:- in swine in the United States Midwest: an emerging multidrug-resistant clade. Clin Infect Dis. 2018;66:877–85. PubMed https://doi.org/10.1093/cid/cix909

12. Croucher NJ, Page AJ, Connor TR, Delaney AJ, Keane JA, Bentley SD, et al. Rapid phylogenetic analysis of large samples of recombinant bacterial whole genome sequences using Gubbins. Nucleic Acids Res. 2015;43:e15. PubMed https://doi.org/10.1093/nar/gku1196

13. Kozlov AM, Darriba D, Flouri T, Morel B, Stamatakis A. RAxML-NG: a fast, scalable and user-friendly tool for maximum likelihood phylogenetic inference. Bioinformatics. 2019;35:4453–5. PubMed https://doi.org/10.1093/bioinformatics/btz305

14. Rambaut A. FigTree. 1.4.4 ed; 2018.

15. Yu G. Using ggtree to visualize data on tree-like structures. Curr Protoc Bioinformatics. 2020;69:e96. PubMed https://doi.org/10.1002/cpbi.96

16. Wang LG, Lam TT, Xu S, Dai Z, Zhou L, Feng T, et al. Treeio: an R package for phylogenetic tree input and output with richly annotated and associated data. Mol Biol Evol. 2020;37:599–603. PubMed https://doi.org/10.1093/molbev/msz240

17. Yu G. Data integration, manipulation and visualization of phylogenetic trees. 1st ed. New York: Chapman and Hall/CRC; 2022.

18. Wickham HA, Averick M, Bryan J, Chang W, McGowan LD, François R, et al. Welcome to the Tidyverse. J Open Source Softw. 2019;4:1686. https://doi.org/10.21105/joss.01686

19. Nurk S, Bankevich A, Antipov D, Gurevich AA, Korobeynikov A, Lapidus A, et al. Assembling single-cell genomes and mini-metagenomes from chimeric MDA products. J Comput Biol. 2013;20:714–37. PubMed https://doi.org/10.1089/cmb.2013.0084

20. Mikheenko A, Prjibelski A, Saveliev V, Antipov D, Gurevich A. Versatile genome assembly evaluation with QUAST-LG. Bioinformatics. 2018;34:i142–50. PubMed https://doi.org/10.1093/bioinformatics/bty266

21. Langmead B, Salzberg SL. Fast gapped-read alignment with Bowtie 2. Nat Methods. 2012;9:357–9. PubMed https://doi.org/10.1038/nmeth.1923

22. Langmead B, Wilks C, Antonescu V, Charles R. Scaling read aligners to hundreds of threads on general-purpose processors. Bioinformatics. 2019;35:421–32. PubMed https://doi.org/10.1093/bioinformatics/bty648

23. Li H. A statistical framework for SNP calling, mutation discovery, association mapping and population genetical parameter estimation from sequencing data. Bioinformatics. 2011;27:2987–93. PubMed https://doi.org/10.1093/bioinformatics/btr509

24. Bushnell B, Rood J, Singer E. BBMerge—accurate paired shotgun read merging via overlap. PLoS One. 2017;12:e0185056. PubMed https://doi.org/10.1371/journal.pone.0185056

25. Feldgarden M, Brover V, Gonzalez-Escalona N, Frye JG, Haendiges J, Haft DH, et al. AMRFinderPlus and the Reference Gene Catalog facilitate examination of the genomic links among antimicrobial resistance, stress response, and virulence. Sci Rep. 2021;11:12728. PubMed https://doi.org/10.1038/s41598-021-91456-0

26. Carattoli A, Zankari E, García-Fernández A, Voldby Larsen M, Lund O, Villa L, et al. In silico detection and typing of plasmids using PlasmidFinder and plasmid multilocus sequence typing. Antimicrob Agents Chemother. 2014;58:3895–903. PubMed https://doi.org/10.1128/AAC.02412-14

27. Altschul SF, Gish W, Miller W, Myers EW, Lipman DJ. Basic local alignment search tool. J Mol Biol. 1990;215:403–10. PubMed https://doi.org/10.1016/S0022-2836(05)80360-2

28. Yoshida CE, Kruczkiewicz P, Laing CR, Lingohr EJ, Gannon VP, Nash JH, et al. The Salmonella In Silico Typing Resource (SISTR): an open web-accessible tool for rapidly typing and subtyping draft *Salmonella* genome assemblies. PLoS One. 2016;11:e0147101. PubMed https://doi.org/10.1371/journal.pone.0147101

29. Jolley KA, Maiden MC. BIGSdb: scalable analysis of bacterial genome variation at the population level. BMC Bioinformatics. 2010;11:595. PubMed https://doi.org/10.1186/1471-2105-11-595

30. Wick RR, Judd LM, Gorrie CL, Holt KE. Unicycler: resolving bacterial genome assemblies from short and long sequencing reads. PLOS Comput Biol. 2017;13:e1005595. PubMed https://doi.org/10.1371/journal.pcbi.1005595

31. Wick RR, Schultz MB, Zobel J, Holt KE. Bandage: interactive visualization of de novo genome assemblies. Bioinformatics. 2015;31:3350–2. PubMed https://doi.org/10.1093/bioinformatics/btv383

32. Clinical and Laboratory Standards Institute. Performance standards for antimicrobial susceptibility testing. 33rd edition. Wayne (PA): The Institute; 2023.

33. Food and Drug Administration. 2018 NARMS update: integrated report summary. 2020 [cited 2023 Jan 7]. https://www.fda.gov/animal-veterinary/national-antimicrobial-resistance-monitoring-system/2018-narms-update-integrated-report-summary

34. R Core Team. R: a language and environment for statistical computing. R Foundation for Statistical Computing. Vienna, Austria; 2022.

35. Abramson JH. WINPEPI updated: computer programs for epidemiologists, and their teaching potential. Epidemiol Perspect Innov. 2011;8:1. PubMed https://doi.org/10.1186/1742-5573-8-1

36. Aviv G, Tsyba K, Steck N, Salmon-Divon M, Cornelius A, Rahav G, et al. A unique megaplasmid contributes to stress tolerance and pathogenicity of an emergent *Salmonella enterica* serovar Infantis strain. Environ Microbiol. 2014;16:977–94. PubMed https://doi.org/10.1111/1462-2920.12351

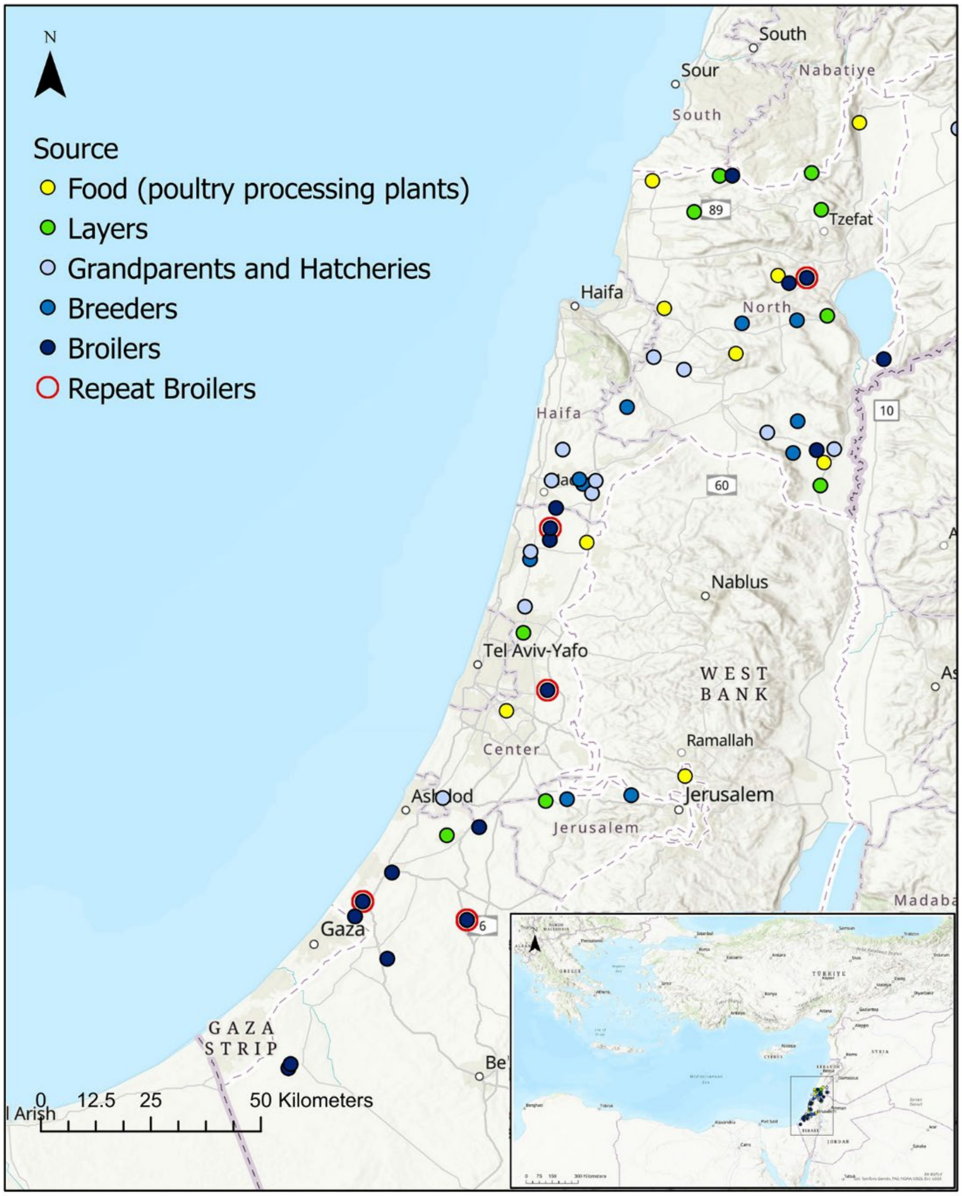

Appendix 1 Figure 1. Map of Israel showing sampling locations of poultry isolates chosen for whole genome sequencing. Insert showing regional context. Isolates from different stages of the broiler production sector including grandparent breeding stock, pullets and hatcheries (n = 12), parent breeders (n = 10) and commercial broilers (n = 38) are shown in shades of blue, poultry processing plants (n = 9, yellow), and layer chicken farms (n = 9, green). Isolates from commercial broiler barns which underwent repeat sampling are shown here marked with a red circle (n = 10).

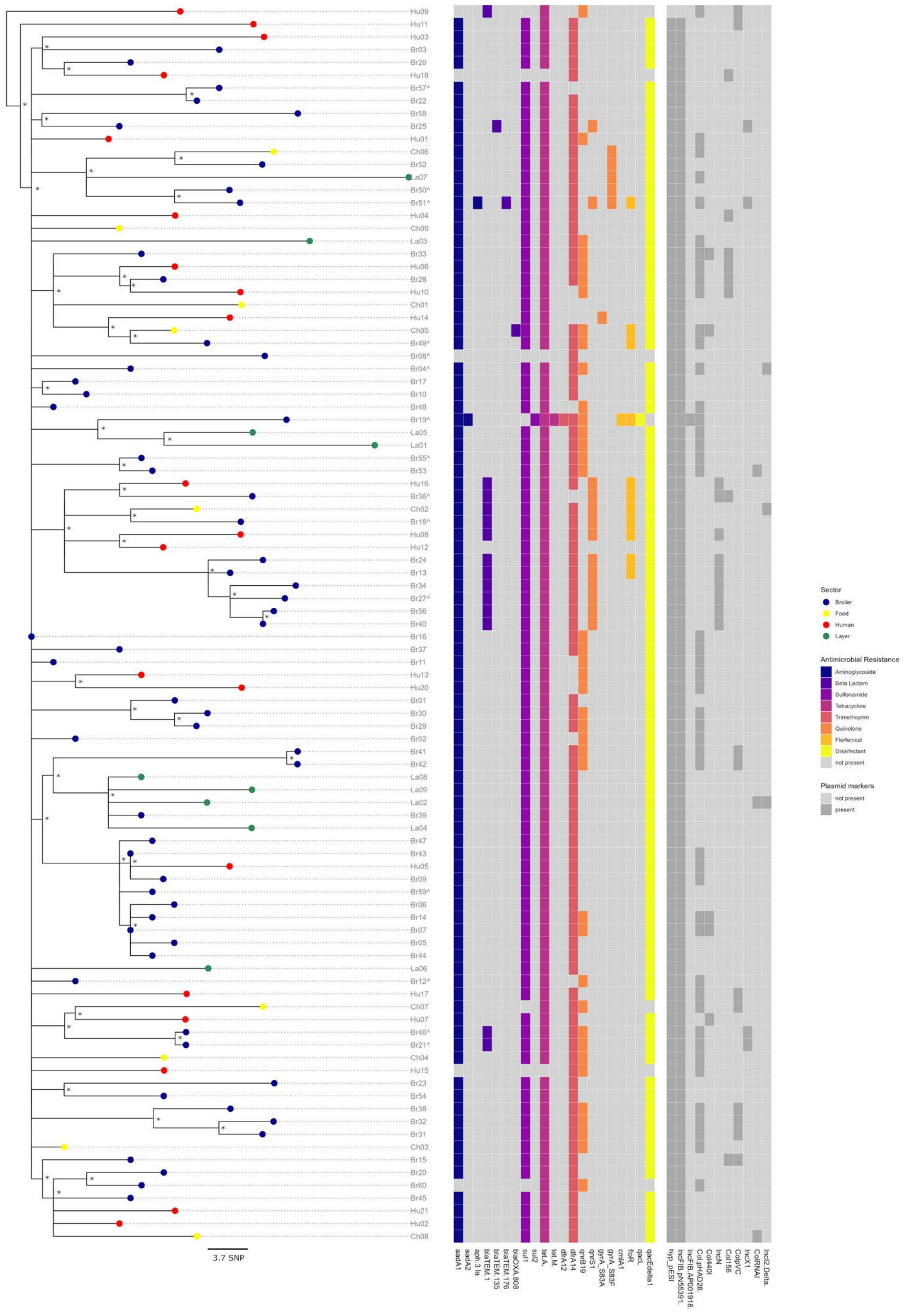

Appendix 1 Figure 2. Maximum-likelihood tree reconstructed using whole-genome sequences of *Salmonella* Muenchen isolates collected from broilers (n = 60), layers (n = 9), food products of poultry origin (whole chicken carcasses sampled in poultry slaughterhouses; n = 9), and humans (n = 21) in Israel during June 2020–January 2022. We used a hybrid assembly of *S.* Muenchen ST82 (Br19) strain as a reference genome. *S.* Muenchen ST112 (SRR6222324) was used to root the tree (not shown). The analysis included 1,227 SNPs in the alignment (0–69 pairwise SNPs, median = 31). Because isolates Hu19 and Br35 were duplicates of isolate Br16, we removed them from the analysis. Tip colors indicate

sector. Asterisks (*) indicate nodes with >70% bootstrap support. Scale bar represents SNP difference. The caret symbol (^) indicates isolates in which antimicrobial sensitivity was tested in vitro (see results in Table 2 and Appendix 2 Table 2). The heatmap includes (i) the presence of acquired antimicrobial resistance genes and point mutations conferring resistance to aminoglycoside, beta lactam, sulfonamide, tetracycline, trimethoprim, quinolone, florfenicol, and disinfectant agents; (ii) the presence of a pESI genetic marker 'hyp_pESI' and the most prevalent plasmid replicons (present in >1 isolate).

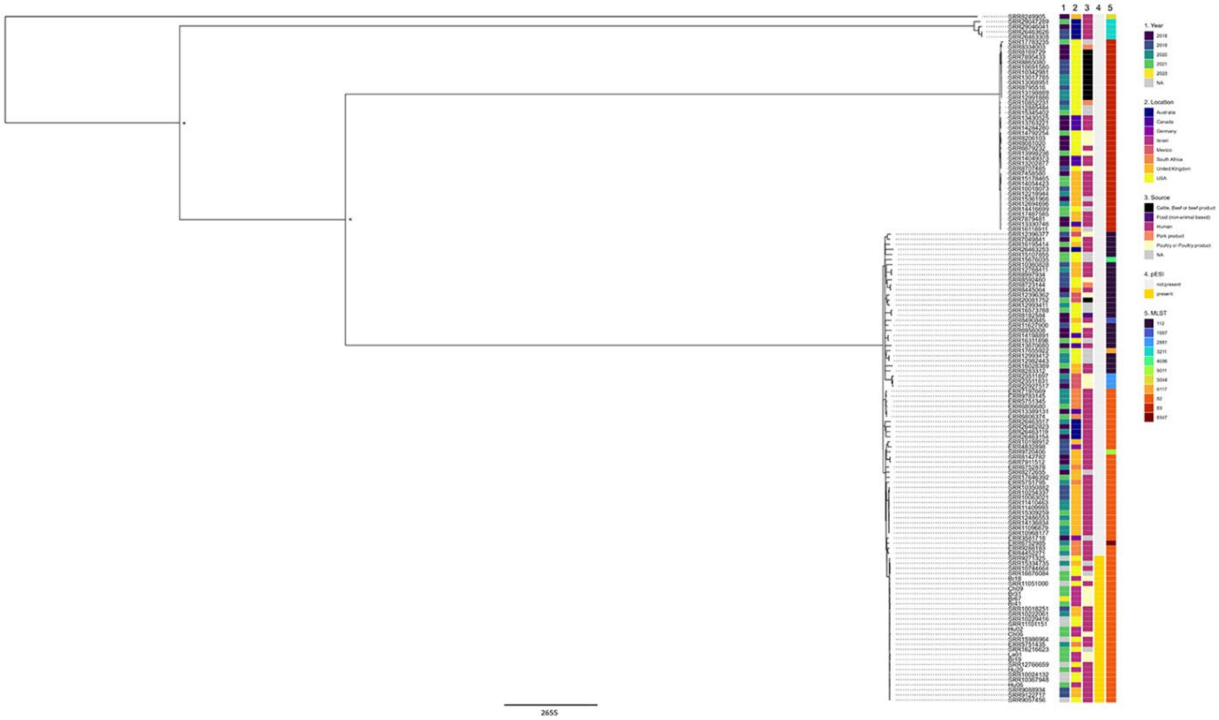

Appendix 1 Figure 3. Maximum-likelihood tree reconstructed using whole-genome sequences (WGS) of a selection of *Salmonella enterica* serovar Muenchen isolates that were chosen to represent the different sources and genetic diversity of *Salmonella* Muenchen in Israel (n = 11) and from publicly available *Salmonella* Muenchen whole-genome sequencing through the National Center for Biotechnology Information (n = 125) in study of *Salmonella* Muenchen in poultry and humans, Israel, June 2020–June 2023. We used a hybrid assembly of *Salmonella* Muenchen sequence type 82 (Br19) strain as a reference genome and used *Salmonella* Braenderup (National Center for Biotechnology Information Sequence Read Archive accession no. SRR5874793) to root the tree (not shown). The analysis included 58,030 single-nucleotide polymorphisms (SNPs) in the alignment (1–26,142 pairwise SNPs, median 394). Asterisks (*) indicate nodes with >70% bootstrap support. Data shown in the heatmap: 1) the year of sample collection, 2) the country of sample collection, 3) the source of the sample, 4) the presence or absence of pESI, and 5) multilocus sequence type. Scale bar represents SNP difference.
